# Rectus femoris stiffness is associated with aerobic capacity in healthy young men: a comparative shear wave elastography study among lower-limb muscles

**DOI:** 10.1186/s12891-026-10438-3

**Published:** 2026-09-04

**Authors:** Hasan Kara, Hüseyin Kaplan, Gizem Cengiz, İsa Cüce, Furkan Sarıoğlu, Hakan İmamoğlu

**Affiliations:** 1 Department of Physical Medicine and Rehabilitation, Erciyes University Faculty of Medicine, Kayseri, Türkiye; 2 Department of Rheumatology, Aksaray Training and Research Hospital, Aksaray, Türkiye; 3 Department of Rheumatology, Kırıkkale Yüksek İhtisas Hospital, Kırıkkale, Türkiye; 4 Department of Radiology, Erciyes University Faculty of Medicine, Kayseri, Türkiye

**Keywords:** Shear wave elastography, Rectus femoris, VO₂max, Soleus, Muscle architecture, Cardiorespiratory fitness, Aerobic capacity

## Abstract

**Background:**

This study aimed to comparatively investigate the relationships between the ultrasonographic architectural and viscoelastic properties of the soleus, medial gastrocnemius (MG), and rectus femoris (RF) muscles and objectively measured aerobic capacity in healthy young adult males.

**Methods:**

This observational cross-sectional study included 59 healthy male medical students aged 18–30 years. The muscle thickness, pennation angle, fascicle length, and shear wave elastography (SWE) stiffness of the right lower-extremity RF, MG, and soleus were measured ultrasonographically. Aerobic capacity was evaluated using cardiopulmonary exercise testing (CPET) with the Bruce protocol, and VO₂max was recorded as the primary outcome. Participants were stratified into low- and high-aerobic capacity groups based on the median VO₂max value (29.4 mL·kg⁻¹·min⁻¹). Associations were examined using correlation analysis and multiple linear regression.

**Results:**

No significant between-group differences were observed in any ultrasonographic or SWE parameter (low vs. high aerobic capacity groups). Conventional architectural parameters (muscle thickness, pennation angle, and fascicle length) showed no significant correlations with VO₂max across all assessed muscles. RF SWE velocity demonstrated a significant positive correlation with VO₂max (*r* = 0.364, *p* = 0.005), designated a priori as the primary comparison; RF Young’s modulus showed a smaller, exploratory correlation (*r* = 0.274, *p* = 0.036) that did not survive multiplicity correction, and no soleus or MG SWE parameters correlated with aerobic capacity. In multiple linear regression analysis, RF SWE velocity (β = 0.280, *p* = 0.021) and BMI (β=−0.555, *p* < 0.001) were independently associated with VO₂max (R²=0.310).

**Conclusions:**

RF shear wave velocity is independently associated with aerobic capacity (VO₂max) in healthy young men, whereas no significant association was detected for the soleus or MG. These associations were not formally compared between muscles.

## Background

Physical activity, defined as any bodily movement produced by skeletal muscles that results in energy expenditure, is a fundamental determinant of health and physical performance [1]. Its beneficial effects on aerobic capacity are mediated not only by the volume of activity performed but also by the structural and mechanical adaptations induced in the locomotor skeletal muscle [2]. These training-induced adaptations ultimately shape an individual’s maximal oxygen uptake (VO₂max), which is widely accepted as the criterion measure of cardiorespiratory fitness and a strong predictor of both athletic performance and long-term health outcomes [3, 4]. At the peripheral level, VO₂max is substantially governed by the structural and mechanical properties of active muscles, which determine the oxygen extraction capacity, force production efficiency, and metabolic output during progressive exercise [3].

Among the lower-extremity muscles that contribute to aerobic performance, the soleus, medial gastrocnemius (MG), and rectus femoris (RF) represent three functionally and metabolically distinct muscle groups, each warranting individual consideration as potential ultrasonographic markers of aerobic capacity. The soleus is composed predominantly of slow-twitch type I fibres, conferring exceptional fatigue resistance and oxidative capacity, and is widely regarded as the cornerstone of aerobic metabolism during sustained low-intensity activity [5–7]. The MG, as part of the triceps surae complex, contributes critically to plantar flexion and push-off mechanics during locomotion, serving as a primary force transmitter between the lower limb and the ground across a wide range of exercise intensities [8–10]. In contrast, the RF is a biarticular muscle that facilitates both hip flexion and knee extension, making it a critical contributor to force generation and power transfer during high-intensity locomotion [11, 12]. These three muscles represent a functional spectrum from oxidative and endurance-oriented to power-generating, making their comparative ultrasonographic evaluation particularly informative. Advances in musculoskeletal ultrasonography have enabled the noninvasive quantification of these distinct structural and viscoelastic properties across multiple muscle groups.

Conventional ultrasonographic parameters (muscle thickness, fascicle length, and pennation angle) provide quantitative insights into muscle architecture and its potential force-generating capacity [13, 14]. Complementing these structural measures, shear wave elastography (SWE) provides a quantitative index of muscle stiffness expressed as Young’s modulus (kPa) or shear wave velocity (m/s) [15], reflecting the viscoelastic behaviour of muscle tissue arising from fibre composition, intramuscular connective tissue content, and architectural organisation [16, 17]. These mechanical properties differ substantially across muscle groups and may be differentially sensitive to aerobic training adaptations [18]. Although a recent study reported associations between conventional architectural parameters and cardiopulmonary exercise testing (CPET)-measured VO₂max in exercising and non-exercising young adults [19], none has simultaneously assessed SWE-derived viscoelastic properties alongside architectural parameters across multiple lower-limb muscles. Furthermore, SWE-derived stiffness has not been evaluated in parallel across multiple lower limb muscles in relation to objectively measured maximal aerobic capacity assessed by CPET.

Therefore, this cross-sectional study aimed to comparatively evaluate the relationships between the ultrasonographic architectural and viscoelastic properties of the soleus, MG, and RF muscles and objectively measured maximal aerobic capacity (VO₂max via CPET) and self-reported physical activity in healthy young adult men.

## Methods

### Study design and participants

This observational cross-sectional study was conducted at the Department of Physical Medicine and Rehabilitation, Erciyes University Faculty of Medicine. A total of 59 healthy male medical students aged 18–30 years were recruited voluntarily. The exclusion criteria included a history of lower extremity trauma within the preceding six months, any known neuromuscular or musculoskeletal disorder, and medical conditions contraindicating CPET. This study was restricted to male participants to minimise the confounding effects of sex-related differences in muscle architecture, body composition, and aerobic capacity. The study protocol was approved by the Erciyes University Clinical Research Ethics Committee (Date: 25.09.2023; Decision No 2023/601), and written informed consent was obtained from all participants prior to enrolment, in accordance with the Declaration of Helsinki.

### Anthropometric measurements and body composition

Demographic characteristics, including age, height, and body weight, were recorded for all participants, and body mass index (BMI) was calculated as weight divided by height squared (kg/m²). Body composition, including total fat mass, fat percentage, total skeletal muscle mass, and segmental lower-extremity lean mass, was assessed using a bioelectrical impedance analysis (BIA) device (InBody 770, Biospace, Seoul, South Korea).

### Self-reported physical activity assessment

Physical activity levels were assessed using the International Physical Activity Questionnaire–Short Form (IPAQ-SF), which captures the frequency and duration of vigorous-intensity, moderate-intensity, and walking activities performed over the preceding seven days. Total physical activity scores were expressed as metabolic equivalent task minutes per week (MET-min/week) using standardised scoring algorithms: walking (3.3 MET × min × days), moderate-intensity activity (4.0 MET × min × days), and vigorous-intensity activity (8.0 MET × min × days) [20, 21].

### Ultrasonographic evaluation

All ultrasonographic examinations were performed on the right lower extremity by a single experienced physiatrist with more than ten years of expertise in musculoskeletal ultrasonography, under the supervision of a radiologist specialising in musculoskeletal elastography, using a GE LOGIQ P8 ultrasound system equipped with a 7–12 MHz linear-array transducer (GE Healthcare, Chicago, IL, USA). All measurements were performed after a minimum rest period of two hours to ensure passive muscle conditions and minimise the effect of prior physical activity on SWE values. All examinations were conducted in a temperature-controlled room (approximately 22–24 °C). Measurements were obtained according to standardised anatomical landmarks to ensure reproducibility.

The RF was assessed with the participant in the supine position with the knee fully extended. The measurement site was the midpoint between the lateral epicondyle of the femur and the anterior superior iliac spine [22]. The MG and soleus muscles were examined with the participant in the prone position and the ankle maintained in a neutral position (90°). The MG was measured at the level of the maximal calf circumference, and the soleus was assessed at the same level at the site of maximum muscle belly prominence in the posterior compartment [22].

**Fig. 1:**
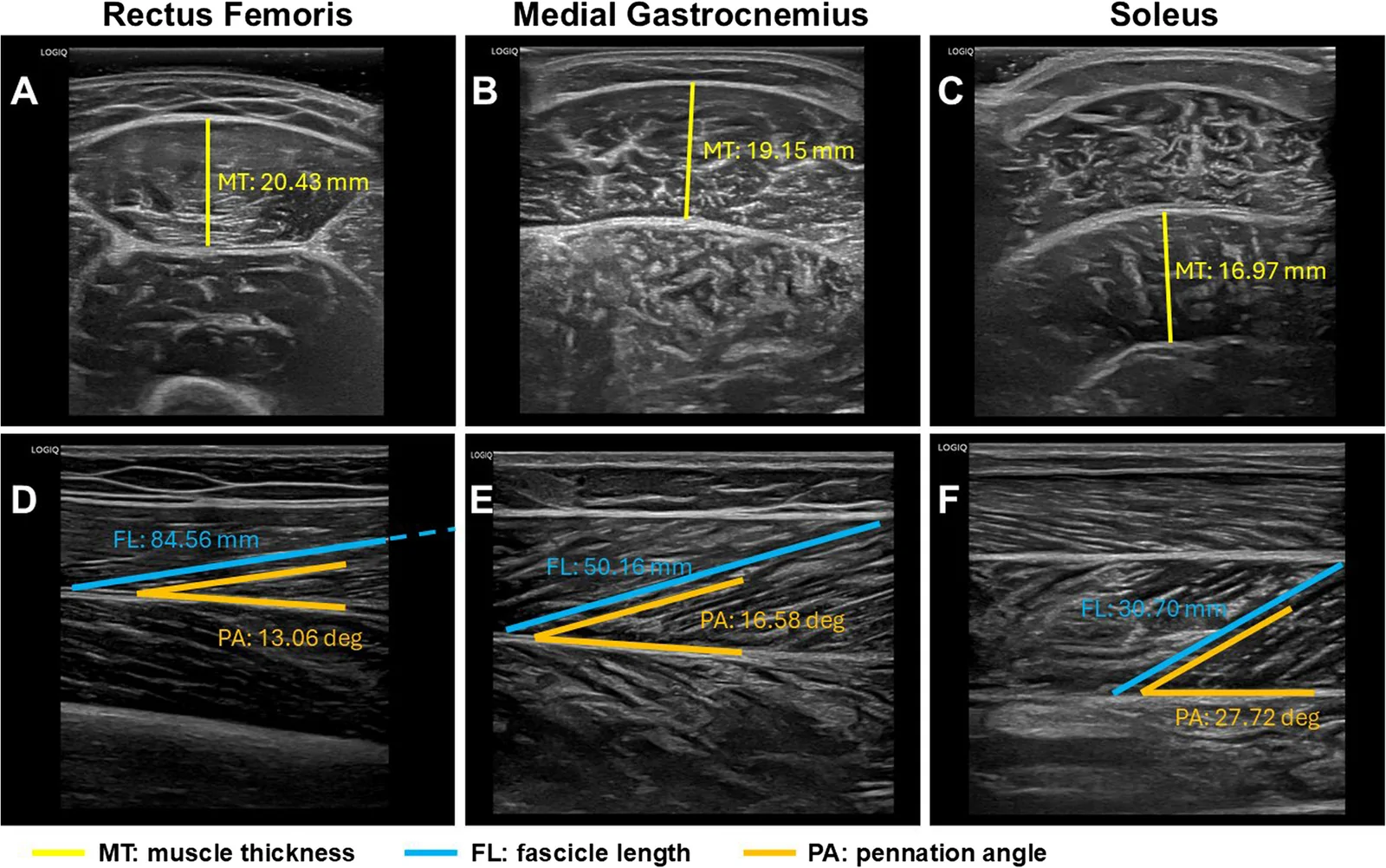
Representative B-mode ultrasonographic images of the RF, MG, and soleus muscles. Panels **A**–**C** show axial (transverse) images used for muscle thickness measurement; yellow lines indicate the distance between the superficial and deep aponeuroses, measured perpendicular to the fascicle plane. Panels **D**–**F** show longitudinal images used for fascicle length and pennation angle measurements; blue lines represent fascicle length traced from the superficial to the deep aponeurosis, and orange lines indicate the pennation angle between the muscle fascicle and the deep aponeurosis

**Fig. 2:**
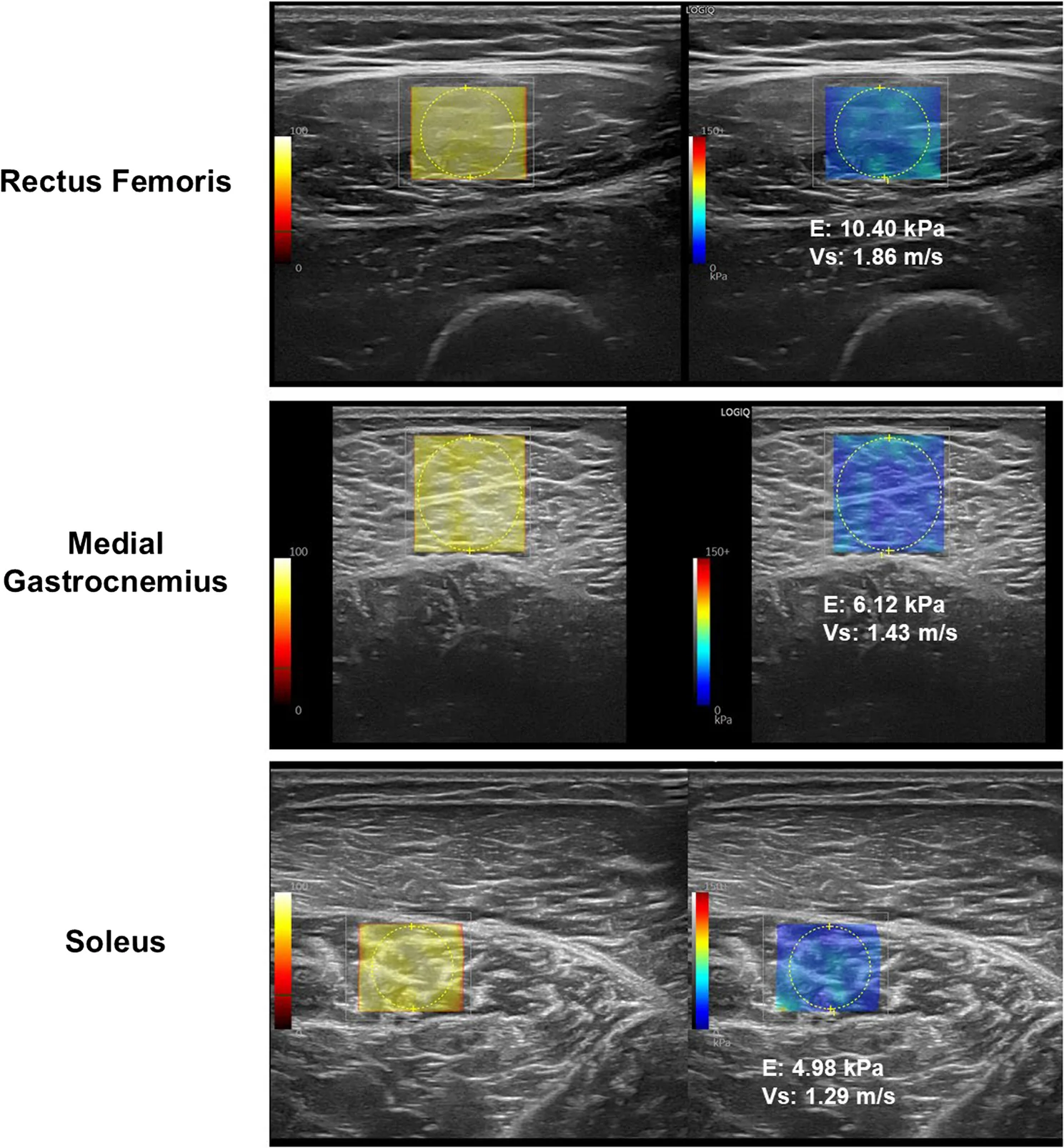
Representative SWE images of the RF, MG, and soleus muscles. Each panel displays the dual-screen format: B-mode reference image (left) with the region of interest (ROI, dashed circle) and the corresponding colour-coded elastography map (right; 0–150 + kPa scale). Mean Young’s modulus (E, kPa) and shear wave velocity (Vs, m/s) are displayed in the lower right of each panel. These values were generated automatically by the ultrasound system and have been re-typed for legibility; the numerical values are unaltered. E, Young’s modulus; Vs, shear wave velocity; kPa, kilopascal

For each muscle, the muscle thickness, fascicle length, and pennation angle were recorded using B-mode imaging. Muscle thickness was measured on axial (transverse) images, whereas fascicle length and pennation angle were assessed on longitudinal images. Each parameter was measured three times, and the mean value was used for the analysis. Representative B-mode images with measurement annotations are presented in Fig. 1. SWE was performed to quantify muscle stiffness, and the results were expressed as Young’s modulus (kPa) and shear wave velocity (m/s). Both parameters were displayed automatically by the ultrasound system; neither was calculated by the investigators. Young’s modulus (E) is not an independently measured quantity but a deterministic function of shear-wave velocity (c), related by E = 3ρc², where ρ is the assumed tissue density (1000 kg/m³) [23]. The two values displayed by the system were consistent with this relationship throughout. As Young’s modulus is a direct function of shear-wave velocity, the two indices represent near-collinear expressions of the same tissue property; both are reported for completeness and comparability with prior studies, and shear-wave velocity, the quantity directly measured by the system, was used for the pre-specified primary analysis. A circular region of interest (ROI) was placed within the muscle belly, avoiding visible fascia, aponeurotic interfaces, and tendinous tissue. Rather than a fixed size, the ROI diameter was adapted for each acquisition to encompass as much of the muscle belly as possible while remaining within a region of adequate elastographic signal quality and excluding fascial, aponeurotic, and tendinous structures. All SWE acquisitions were performed in the transverse (axial) plane. SWE measurements were obtained from three separate acquisitions for each muscle, and the median value was used for analysis. Representative SWE images for each muscle are presented in Fig. 2. To minimise pressure-induced artificial stiffness elevation, a generous coupling gel was applied, and the transducer contact pressure was kept minimal throughout all SWE acquisitions.

### Exercise capacity assessment

Functional exercise capacity was assessed using the Six-Minute Walk Test (6 MWT), which was conducted in a 30-meter indoor corridor in accordance with established guidelines. Participants were instructed to walk as far as possible during the six-minute period, and the total distance covered (m) was recorded [24].

Maximal aerobic capacity was assessed using CPET on a motorised treadmill with the standard Bruce protocol [25]. Gas exchange variables were measured breath-by-breath using a calibrated metabolic cart (Vyntus CPX; Vyaire Medical, Höchberg, Germany). The primary outcome, VO₂max, was defined as the highest 30-second-averaged oxygen uptake at which a plateau in oxygen consumption was observed despite an increasing workload, as evidenced by an increase of less than 2.1 mL·kg⁻¹·min⁻¹ between consecutive stages. To confirm maximal effort, testing was continued until volitional exhaustion or until the following criteria were met: respiratory exchange ratio (RER) ≥ 1.10 and/or achievement of ≥ 90% of age-predicted maximal heart rate. Heart rate was continuously monitored, and perceived exertion was assessed using the Borg scale [26, 27]. All tests were supervised by trained personnel, and standard safety and termination criteria were applied throughout the study. VO₂max was expressed in body-mass-normalised units (mL·kg⁻¹·min⁻¹) for the primary analysis and in absolute units (mL·min⁻¹) for the sensitivity analysis; both represent the same measured plateau in oxygen consumption, differing only in unit expression.

### Statistical analysis

Statistical analyses were performed using SPSS for Windows version 26.0 (IBM Corp., Armonk, NY, USA). Data normality was assessed using the Shapiro–Wilk test and visual inspection of histograms and quantile–quantile plots. Descriptive statistics are presented as mean ± standard deviation for normally distributed variables and median (interquartile range) for non-normally distributed variables. Categorical variables were expressed as frequencies and percentages.

For exploratory between-group comparisons, participants were dichotomised into low and high aerobic capacity groups based on the median VO₂max value (29.4 mL·kg⁻¹·min⁻¹), given the homogeneous aerobic capacity profile of the study population, which precluded the application of established normative values. Between-group differences were evaluated using the independent-samples t-test or Mann–Whitney U test, as appropriate.

Associations between ultrasonographic parameters and exercise capacity indices (VO₂max, 6 MWT distance) and physical activity (IPAQ total MET-min/week) were examined using Pearson’s or Spearman’s correlation coefficients, depending on the data distribution. The RF shear-wave velocity–VO₂max association was pre-specified a priori as the primary comparison, on mechanistic grounds (the biarticular, power-generating role of the RF) and its consistency with prior work [28]; all remaining correlations (soleus and MG parameters, and all 6 MWT and IPAQ associations) were treated as exploratory. For the exploratory correlations, the false discovery rate (FDR) was controlled using the Benjamini-Hochberg procedure, and both unadjusted (p) and FDR-adjusted (q) values are reported (Table 3).

Multiple linear regression analysis (Enter method) was performed to identify variables independently associated with VO₂max, with all candidate variables entered simultaneously into the model. Multicollinearity was assessed using the variance inflation factor (VIF), with a VIF > 10 considered indicative of problematic collinearity. The model assumptions, including residual normality and homoscedasticity, were verified. A two-tailed p-value of < 0.05 was considered statistically significant.

## Results

A total of 59 healthy male medical students were included in this analysis. The mean age of the cohort was 23.2 ± 1.14 years. Based on the median VO₂max value (29.4 mL·kg⁻¹·min⁻¹), the participants were categorised into low (*n* = 30) and high (*n* = 29) aerobic capacity groups. Demographic and clinical characteristics are presented in Table 1. No significant differences were observed between the groups in terms of age or self-reported physical activity levels (IPAQ total MET-min/week) (*p* > 0.05). Participants in the low VO₂max group had significantly higher body weight, height, BMI, fat mass, and body fat percentage than those in the high VO₂max group (*p* < 0.05 for all). In contrast, total skeletal muscle mass and skeletal muscle index (SMI) did not differ between the groups (*p* > 0.05). Functional exercise capacity assessed using the 6 MWT was significantly greater in the high VO₂max group (*p* = 0.001).

**Table 1:** Demographic and clinical characteristics of the participants according to aerobic capacity

Variables	Total (*n* = 59)	Low VO_2_max group (*n* = 30)	High VO_2_max group (*n* = 29)	*p*
Age (years)	23.2 ± 1.14	23.2 ± 1.28	23.1 ± 0.99	0.962
Body weight (kg)	78.2 ± 13.7	82.48 ± 15.85	73.53 ± 8.87	**0.011**
Height (cm)	178 ± 5.94	179.2 ± 5.63	176.6 ± 6.06	**0.037**
BMI (kg/m²)	24.6 ± 3.73	25.65 ± 4.50	23.36 ± 2.13	**0.017**
IPAQ (MET-min/week)	2045 ± 1444	2048 ± 1414	2042 ± 1503	0.909
6MWT distance (m)	707 ± 45.5	689.2 ± 43.21	726.2 ± 40.44	**0.001**
VO_2_max mL·kg⁻¹·min⁻¹	30.3 ± 5.21	26.4 ± 2.51	34.7 ± 3.74	**< 0.001**
Fat mass (kg)	16.7 ± 9.78	20.04 ± 11.50	12.91 ± 5.56	**0.034**
Skeletal muscle mass (kg)	34.9 ± 4.26	35.32 ± 4.49	34.36 ± 4.02	0.255
Body fat percentage (%)	20.2 ± 8.60	22.91 ± 9.62	17.23 ± 6.18	**0.010**
SMI (kg/m^2^)	8.22 ± 0.63	8.29 ± 0.66	8.15 ± 0.59	0.407

Values are presented as the mean ± standard deviation. Participants were categorised into low and high aerobic capacity groups based on the median VO₂max value (29.4 mL·kg⁻¹·min⁻¹). *BMI* Body mass index, *IPAQ* International Physical Activity Questionnaire (short form), *MET-min/week* Metabolic equivalent task minutes per week, *6 MWT* 6-minute walk test, *VO₂max* Maximal oxygen uptake, *SMI* Skeletal muscle index. *p* values indicate between-group comparisons

The between-group comparisons of the right lower extremity ultrasonographic and SWE elastography parameters are presented in Table 2. No significant differences were observed between the low and high VO₂max groups in the muscle thickness, pennation angle, or fascicle length of the soleus, MG, or RF muscles (all *p* > 0.05). Similarly, the SWE-derived stiffness parameters, expressed as both Young’s modulus (kPa) and shear wave velocity (m/s), were comparable between groups across all assessed muscles (all *p* > 0.05). Although the RF Young’s modulus was numerically higher in the high VO₂max group (17.91 ± 5.74 kPa vs. 15.63 ± 4.09 kPa), this difference did not reach statistical significance (*p* = 0.081).

**Table 2:** Between-group comparison of ultrasonographic and SWE findings

Variables	Low VO_2_max group (*n* = 30)	High VO_2_max group (*n* = 29)	*p*
Soleus			
Muscle thickness (mm)	18.32 ± 3.99	17.39 ± 2.85	0.309
SWE (kPa)	9.83 ± 4.63	7.99 ± 2.26	0.205
SWE velocity (m/s)	1.77 ± 0.39	1.62 ± 0.23	0.210
Pennation angle (°)	29.93 ± 8.25	28.14 ± 6.12	0.353
Fascicle length (mm)	38.16 ± 11.70	35.09 ± 8.00	0.430
Medial Gastrocnemius			
Muscle thickness (mm)	18.82 ± 2.66	18.87 ± 2.48	0.938
SWE (kPa)	13.65 ± 8.71	11.71 ± 8.73	0.255
SWE velocity (m/s)	2.04 ± 0.629	1.88 ± 0.617	0.268
Pennation angle (°)	23.79 ± 3.68	23.42 ± 3.16	0.683
Fascicle length (mm)	49.00 ± 7.99	49.75 ± 8.80	0.734
Rectus Femoris			
Muscle thickness (mm)	24.80 ± 3.81	25.03 ± 4.10	0.827
SWE (kPa)	15.63 ± 4.09	17.91 ± 5.74	0.081
SWE velocity (m/s)	2.27 ± 0.30	2.41 ± 0.39	0.115
Pennation angle (°)	12.82 ± 4.08	13.31 ± 3.26	0.529
Fascicle length (mm)	124.76 ± 41.25	113.28 ± 26.11	0.212

Values are presented as mean ± standard deviation and reflect the measurements obtained from the right lower extremity. *SWE* Shear wave elastography, *kPa* Kilopascal (Young’s modulus); *p*-values indicate between-group comparisons

In the primary, pre-specified analysis, VO₂max demonstrated a significant moderate positive correlation with RF shear-wave velocity (*r* = 0.364, *p* = 0.005), which was designated a priori as the primary comparison and, unlike the exploratory correlations, was reported without multiplicity adjustment. Among the exploratory correlations, RF Young’s modulus showed a smaller positive correlation with VO₂max (*r* = 0.274, *p* = 0.036), 6 MWT distance was negatively correlated with the soleus pennation angle (*r* = − 0.272, *p* = 0.037), and self-reported physical activity (IPAQ total MET-min/week) was positively correlated with MG stiffness assessed using both Young’s modulus (*r* = 0.308, *p* = 0.018) and shear-wave velocity (*r* = 0.312, *p* = 0.016); however, none of these exploratory associations retained significance after Benjamini-Hochberg correction (all q > 0.05). No other ultrasonographic or SWE parameter was significantly correlated with VO₂max, 6 MWT distance, or IPAQ scores at the unadjusted level (all *p* > 0.05). Both unadjusted (p) and FDR-adjusted (q) values are presented in Table 3.

**Table 3:** Correlations between lower-extremity ultrasonographic parameters and exercise capacity, functional performance, and physical activity indices

Variable	VO_2_max			6MWT distance			IPAQ		
	*r*	*p*	q	*r*	*p*	q	*r*	*p*	q
Soleus									
Muscle thickness (mm)	0.032	0.808	0.919	-0.025	0.853	0.919	-0.139	0.294	0.631
SWE (kPa)	-0.186	0.159	0.631	-0.129	0.330	0.631	0.025	0.850	0.919
SWE velocity (m/s)	-0.180	0.173	0.631	-0.122	0.357	0.654	0.024	0.856	0.919
Pennation angle (°)	-0.213	0.106	0.631	**-0.272**	**0.037**	0.407	-0.147	0.267	0.631
Fascicle length (mm)	-0.018	0.893	0.936	0.073	0.584	0.831	0.027	0.837	0.919
Medial Gastrocnemius									
Muscle thickness (mm)	-0.068	0.611	0.831	-0.165	0.213	0.631	-0.130	0.325	0.631
SWE (kPa)	-0.171	0.196	0.631	-0.155	0.242	0.631	**0.308**	**0.018**	0.396
SWE velocity (m/s)	-0.167	0.207	0.631	-0.154	0.245	0.631	**0.312**	**0.016**	0.396
Pennation angle (°)	-0.212	0.107	0.631	-0.157	0.236	0.631	0.065	0.623	0.831
Fascicle length (mm)	0.109	0.413	0.699	0.138	0.299	0.631	0.070	0.598	0.831
RF									
Muscle thickness (mm)	0.059	0.656	0.849	-0.005	0.971	0.994	-0.051	0.700	0.880
SWE (kPa)	**0.274**	**0.036**	0.407	0.094	0.478	0.779	0.071	0.594	0.831
SWE velocity (m/s)	**0.364**	**0.005**	— ᵃ	0.136	0.306	0.631	0.068	0.608	0.831
Pennation angle (°)	0.001	0.995	0.995	-0.147	0.267	0.631	-0.152	0.250	0.631
Fascicle length (mm)	-0.114	0.389	0.685	-0.027	0.839	0.919	0.208	0.113	0.631

Pearson’s or Spearman’s correlation coefficients were used, depending on the data distribution. *VO₂max* Maximal oxygen uptake, *6 MWT* Six-minute walk test, *IPAQ* International Physical Activity Questionnaire short form, *SWE* Shear wave elastography, *kPa* Kilopascal (Young’s modulus). *p* = unadjusted; q = Benjamini-Hochberg FDR-adjusted *p*-value across the 44 exploratory correlations. Bold indicates *p* < 0.05 before multiplicity adjustment. ᵃ The RF shear-wave velocity–VO₂max correlation was pre-specified as the primary comparison and is reported without multiplicity adjustment

A multiple linear regression analysis using the Enter method was performed to identify variables independently associated with VO₂max. The overall model was statistically significant (*R* = 0.557; R²=0.310; adjusted R²=0.259; F(4,54) = 6.07; *p* < 0.001), explaining 31.0% of the variance in VO₂max. RF shear wave velocity was independently and positively associated with VO₂max (B = 4.179; β = 0.280; *p* = 0.021), whereas BMI was independently and negatively associated with VO₂max (B = − 0.776; β=−0.555; *p* < 0.001). SMI showed a borderline association (β = 0.280; *p* = 0.053), and IPAQ total MET-min/week was not independently associated with VO₂max (*p* = 0.298). The full regression model results are presented in Table 4.

**Table 4:** Multiple linear regression model for VO₂max

Variable	B	SE	β	t	*p*	95% CI for B	VIF
Intercept	21.424	9.731	—	2.20	0.032	1.915–40.933	—
RF SWE velocity (m/s)	4.179	1.755	0.280	2.38	**0.021**	0.660–7.697	1.08
BMI (kg/m²)	−0.776	0.208	−0.555	−3.72	**< 0.001**	−1.194 – −0.358	1.74
SMI (kg/m²)	2.326	1.178	0.280	1.98	0.053	−0.034–4.687	1.58
IPAQ (MET min/week)	−4.48e⁻⁴	4.26e⁻⁴	−0.124	−1.05	0.298	−0.001–4.07e⁻⁴	1.09

Dependent variable: VO₂max (mL·kg⁻¹·min⁻¹). Enter method: all variables were entered simultaneously. Bold *p*-values indicate statistical significance (*p* < 0.05). Model fit: *R* = 0.557; R²=0.310; adjusted R²=0.259; F(4,54) = 6.07; *p* < 0.001. *RF* Rectus femoris, *SWE* Shear wave elastography, *BMI* Body mass index, *SMI* Skeletal muscle index, *IPAQ* International Physical Activity Questionnaire, *VIF* Variance inflation factor

In a sensitivity analysis using absolute VO₂max (mL·min⁻¹) as the dependent variable, the overall model was statistically significant (*R* = 0.615; R²=0.379; adjusted R²=0.333; F(4,54) = 8.23; *p* < 0.001), explaining 37.9% of the variance. SMI showed the strongest independent association (β = 0.515, *p* < 0.001), whereas RF SWE velocity remained significantly and positively associated with absolute VO₂max (β = 0.239, *p* = 0.037). BMI was not independently associated with absolute VO₂max in this model (*p* = 0.396). The full results are presented in Table 5.

**Table 5:** Multiple linear regression model for absolute VO₂max (mL·min⁻¹) (sensitivity analysis)

Variable	B	SE	β	t	*p*	95% CI	VIF
Constant	−1534.318	765.146	—	−2.005	0.050	−3068.344 – −0.292	—
RF SWE velocity (m/s)	295.915	138.001	0.239	2.144	**0.037**	19.240–572.589	1.08
BMI (kg/m²)	14.018	16.389	0.121	0.855	0.396	−18.840–46.876	1.74
SMI (kg/m²)	354.123	92.593	0.515	3.825	**< 0.001**	168.486–539.761	1.58
IPAQ (MET-min/week)	−0.037	0.034	−0.124	−1.103	0.275	−0.104–0.030	1.09

Dependent variable: Absolute VO₂max (mL·min⁻¹). *R* = 0.615; R²=0.379; Adjusted R²=0.333; F(4,54) = 8.23; *p* < 0.001. Cook’s distance max = 0.214; Shapiro–Wilk *p* = 0.373. Bold *p*-values indicate statistical significance (*p* < 0.05). *RF* Rectus femoris, *SWE* Shear wave elastography, *BMI* Body mass index, *SMI* Skeletal muscle index, *IPAQ* International Physical Activity Questionnaire, *VIF* Variance inflation factor, *CI* Confidence interval

## Discussion

This cross-sectional study investigated the relationship between ultrasonographic architectural and viscoelastic properties of key lower-limb muscles and aerobic capacity, as measured by CPET, in healthy young male adults. Although no significant between-group differences were observed in any ultrasonographic or SWE parameter when participants were stratified by median VO₂max, correlation and regression analyses of the full continuous dataset revealed meaningful associations. Conventional architectural parameters showed no significant associations with aerobic capacity across all assessed muscles. Among SWE-derived parameters, only the pre-specified primary association, RF shear-wave velocity with VO₂max (*p* = 0.005), was designated a priori and reported without multiplicity adjustment; none of the exploratory correlations, including those of RF Young’s modulus with VO₂max, soleus pennation angle with 6 MWT distance, and MG stiffness with IPAQ, retained significance after Benjamini-Hochberg correction (all q > 0.05). In the regression analyses, RF SWE velocity and BMI were independently associated with VO₂max.

The significant and independent association between RF SWE velocity and VO₂max may be explained by the unique biomechanical role of the RF as a biarticular muscle facilitating both hip flexion and knee extension, which are critical contributors to force generation and power transfer during high-intensity locomotion [11, 12]. Unlike the soleus and gastrocnemius, which primarily contribute to plantar flexion and postural stabilisation, the RF may be more directly involved in the mechanical demands of progressive maximal exercise [29]. The higher shear wave velocity observed in individuals with greater aerobic capacity (i.e., a stiffer RF was associated with higher VO₂max) may reflect a more organised myofibrillar architecture and greater intramuscular connective tissue stiffness [16, 17]. These findings are broadly consistent with those of a previous myotonometric study in male triathletes, in which RF elasticity and stiffness in both relaxed and contracted states were significantly associated with VO₂max, with RF mechanical properties explaining approximately one-third of the variance in maximal aerobic capacity [28], a proportion strikingly similar to the 31.0% variance explained by our regression model. Importantly, SWE and myotonometry capture distinct mechanical properties: SWE quantifies viscoelastic behaviour at the tissue level through shear wave propagation, whereas myotonometry assesses the surface mechanical response to external oscillation [15, 16, 30]. The independent replication of the RF–VO₂max association across these methodologically distinct modalities and populations that differ substantially in aerobic fitness level strengthens the argument that RF mechanical behaviour reflects physiologically meaningful aspects of maximal aerobic performance.

One notable finding of this study was the absence of a significant association between soleus ultrasonographic parameters and aerobic capacity indices, despite the well-established oxidative profile of this muscle group. These observations are consistent with those of the previous studies. Akkoc et al. [22], studying adolescent female basketball players using the same SWE methodology, found no significant correlations between soleus, gastrocnemius, or RF passive stiffness and VO₂max estimated by the shuttle run test. Similarly, Konrad et al. [8], who examined the passive compression stiffness of the triceps surae and quadriceps in trained male runners and triathletes, reported no significant association between gastrocnemius or RF compression stiffness and oxygen cost during submaximal running. The paradox of the remarkable oxidative phenotype of the soleus muscle alongside its absence of association with VO₂max may be further understood in light of recent experimental evidence. Hamilton et al. [6] demonstrated that the soleus, despite comprising only approximately 1% of body mass, can sustain exceptionally high local rates of oxidative metabolism for hours during low-intensity isolated contractile activity, substantially improving systemic glucose and lipid regulation. This underscores the metabolic superiority of the soleus under submaximal, endurance-oriented conditions. However, this same phenotype may render the soleus relatively insensitive as an ultrasonographic marker of maximal aerobic capacity: VO₂max testing recruits large muscle masses under progressive high-intensity loading, a context fundamentally distinct from the isolated, low-threshold contractile conditions under which the soleus metabolic dominance manifests. Additionally, the homogeneous aerobic capacity profile of our population may have limited inter-individual variation in soleus architecture and stiffness, and SWE measurements in deeper posterior compartment muscles are inherently more susceptible to measurement variability than in superficial muscles such as the RF, both of which may contribute to the observed null finding. Taken together, while the soleus is indeed a ‘metabolic giant’, its structural and viscoelastic parameters were not associated with maximal aerobic performance in a healthy young cohort, highlighting that the oxidative profile alone does not guarantee ultrasonographic utility as a fitness marker.

The findings of studies examining muscle architectural properties show considerable variability. Studies in elite cyclists have reported strong correlations between vastus lateralis thickness and anaerobic power output, while investigations in swimmers have demonstrated sport-specific differences in gastrocnemius fascicle length compared with soccer players [31, 32]. Similarly, the fascicle lengths of the gluteus maximus and gastrocnemius lateralis were significantly greater in sprinters than in long-distance runners [33, 34]. This heterogeneity likely reflects differences in sport discipline, training subcategory, and experience level across study populations [19]. Furthermore, studies directly examining the relationship between ultrasonographically assessed muscle architecture and CPET-measured VO₂max in nonathletic populations remain scarce. In this context, Dağ et al. [19] reported positive correlations between VO₂max and muscle thickness of the RF, vastus intermedius, vastus lateralis, and tibialis anterior, as well as the vastus intermedius pennation angle and tibialis anterior fascicle length in exercising and non-exercising young adults. No such associations were observed in the present study, likely reflecting the narrower range of aerobic capacity and architectural variation of our cohort compared with the design contrasting the exercising and sedentary groups. Critically, Dağ et al. [19] did not include SWE assessment, whereas RF shear wave velocity was significantly and independently associated with VO₂max in the present study. This distinction highlights the complementary informational value of SWE over conventional architectural parameters. Whereas muscle thickness, pennation angle, and fascicle length primarily reflect muscle geometry and gross morphology, SWE quantifies the viscoelastic behaviour of muscle tissue, encompassing fibre composition, intramuscular connective tissue organisation, and myofibrillar structural integrity of the muscle. These properties may be more sensitive to the specific mechanical demands of maximal aerobic exercise.

Participants in the low VO₂max group exhibited significantly higher fat mass and body fat percentage, whereas skeletal muscle mass and SMI did not differ between the groups. This dissociation is physiologically meaningful; when VO₂max is expressed relative to body mass, excess adipose tissue penalises the denominator without contributing to oxygen uptake, thereby disproportionately reducing the normalised aerobic capacity [35]. The comparable self-reported physical activity levels between groups further suggest that these adiposity differences were not simply a reflection of differential activity habits, but rather inter-individual variation in diet, energy balance, or habitual distribution of exercise intensity not captured by the IPAQ-SF. This interpretation is supported by the regression findings, in which BMI displayed the strongest independent negative association with VO₂max. The complementary roles of muscle mass and muscle quality were further illustrated in the sensitivity analysis: SMI showed the strongest independent association with absolute VO₂max, whereas BMI was no longer independently associated, consistent with the notion that absolute oxygen uptake is primarily determined by metabolically active muscle tissue, independent of body mass [35]. Complementing these findings, RF shear wave velocity was independently associated with body-mass-normalised VO₂max even after adjusting for BMI and skeletal muscle mass, suggesting that muscle viscoelastic quality captures a dimension of aerobic performance that is distinct from both adiposity and muscle quantity.

This study has several limitations. The cross-sectional design precludes causal inference, and it remains unclear whether higher aerobic capacity leads to greater RF stiffness through training-induced adaptations or whether a specific muscle mechanical phenotype predisposes individuals to superior aerobic performance. The study population comprised exclusively healthy young male medical students, limiting the generalisability of the results to females, older adults, athletes, and clinical groups. All SWE measurements were obtained under passive resting conditions; dynamic or contraction state stiffness was not assessed, and future studies should examine whether active SWE parameters exhibit stronger associations with aerobic performance. SWE measurements are sensitive to probe pressure, joint position, and hydration status, and residual measurement variability, particularly in deeper posterior compartment muscles, cannot be excluded. Only the right lower extremity was assessed; potential bilateral asymmetry and dominant-limb effects were not examined. Furthermore, although a single primary comparison was pre-specified and the false discovery rate was controlled across the exploratory correlations, the number of exploratory tests means that the possibility of type I error cannot be fully excluded, and the exploratory associations require confirmation in independent samples. Finally, larger studies are needed to confirm these findings and establish normative SWE thresholds for performance stratification in young adults.

## Conclusions

In conclusion, RF viscoelastic properties assessed by SWE were independently associated with aerobic capacity in healthy young men, an association that may reflect the muscle’s biarticular, power-generating role during high-intensity exercise. In contrast, no significant association was detected between soleus or MG ultrasonographic parameters and any aerobic capacity index. The strength of these associations was not formally compared between muscles, and establishing a between-muscle difference would require dedicated statistical testing. Future longitudinal studies should examine whether training interventions selectively modify RF viscoelastic properties, whether active SWE measurements strengthen these associations, and whether SWE may serve as a practical non-invasive tool for fitness screening, training monitoring, and individualised exercise prescription in young adults.

## Data Availability

The datasets generated and/or analysed during the current study are available from the corresponding author on reasonable request.

## Abbreviations

6MWT: 6-minute walk test
BIA: Bioelectrical impedance analysis
BMI: Body mass index
CI: Confidence interval
CPET: Cardiopulmonary exercise testing
FDR: False discovery rate
IPAQ-SF: International Physical Activity Questionnaire–Short Form
MET: Metabolic equivalent task
MG: Medial gastrocnemius
RER: Respiratory exchange ratio
RF: Rectus femoris
ROI: Region of interest
SMI: Skeletal muscle index
SWE: Shear wave elastography
VIF: Variance inflation factor
VO₂max: Maximal oxygen uptake
